# Effect of Cognitive Behavioral Therapy on Improving Anxiety, Depression, and Quality of Life in Pre-Diagnosed Lung Cancer Patients

**DOI:** 10.31557/APJCP.2021.22.11.3455

**Published:** 2021-11

**Authors:** Yusup Subagio Sutanto, Dede Ibrahim, Debree Septiawan, Aris Sudiyanto, Hendra Kurniawan

**Affiliations:** 1 *Department of Pulmonology and Respiratory Medicine, University of Sebelas Maret, Surakarta, Central Java, 57126, Indonesia. *; 2 *Dr Moewardi General Hospital, Surakarta, Central Java, 57126, Indonesia. *; 3 *Department of Psychiatry, University of Sebelas Maret, Surakarta, Central Java, 57126, Indonesia. *; 4 *Department of Public Health and Community Medicine, University of Syiah Kuala, Aceh, 23111, Indonesia. *

**Keywords:** Anxiety, cognitive behavior therapy, depression, lung cancer, quality of life

## Abstract

**Objective:**

To evaluate the effectiveness of cognitive behavior therapy on anxiety, depression, and quality of life of pre-diagnosed lung cancer patients.

**Methods:**

A total of 32 pre-diagnosed subjects were divided into 16 Cognitive behaviour theraphy (CBT)-intervention patients and 16 control subjects. The study subjects were pre-diagnosed lung cancer patients hospitalized at Regional Public Hospital Dr. Moewardi Surakarta. For the treatment group, CBT psychotherapy interventions were given for up to 6 sessions every 2 days. The patient was tested for Hamilton Anxiety Rating Scale (HARS)-based anxiety symptom criteria and Hamilton Depression Rating Scale (HRSD)-based depression and followed The World Health Organization Quality of Life- Brief version (WHOQOL-BREF)-based quality of life criteria. The effect of CBT intervention was measured using an independent t-test and the Mann–Whitney test.

**Results:**

There was a significant difference in the intervention group post-test: HARS criteria decreased by −8.38 ± 2.90, HRSD decreased by an average of −6.75 ± 3.30, and WHOQOL-BREF increased by an average of 16.80 ± 10.13 compared with the control group.

**Conclusion:**

CBT affects the improvement of anxiety, depression, and quality of life for pre-diagnosed lung cancer patients.

## Introduction

Cancer is a serious and potentially life-threatening disease with negative physical and psychological effects on patients. Long-term cancer treatment and the side effects of chemotherapy may also have psychological effects on patients. The side effects of chemotherapy and the cancer itself will decrease the quality of life of the patients (Viriyasiri et al., 2020). Cancer patients usually find it difficult to accept the fact that they have cancer, and this affects their emotional state in the treatment of cancer. Some cancer patients develop mental health problems such as anxiety, depression, post-traumatic stress disorder, adjustment disorders, social phobia, and organic mental disorders (Yi and Syrjala, 2017).

Lung cancer is the most common cause of cancer death worldwide. Every year, the number of deaths from lung cancer is higher than that from breast, colon, and prostate cancer. According to the World Health Organization’s 2017 report, Lung cancer is a leading cause of 1.69 million cancer deaths. In the United States, there were 213,380 new cases of lung cancer in 2007, with around 160,390 deaths. Lung cancer is the most common case of men and the fourth most common case in women, and is the main cause of death based on the results of a hundred-hospital-based research study in Jakarta (Wahidin et al., 2012).

There are two important aspects of lung cancer: survival of patients and quality of life. About 5 years after diagnosis, as many as 17.7% of patients with lung cancer will survive. To date, treatment for lung cancer has focused on reducing incidence and mortality. The quality of life of patients with lung cancer receives less publicity. For patients with lung cancer and risk factors for psychological aspects, an initial assessment is required. Comprehensive treatment of both medically induced lung cancer and clinical therapy improves patients with lung cancer survival. Depression and anxiety are the most common psychiatric aspects of lung cancer patients (Yang, 2009).

Lung cancer patients are very emotionally depressed. In epidemiological studies, patients with lung cancer are reported to have the highest levels of anxiety and depression after diagnosis. Feelings of guilt and stigma due to illness with one’s smoking behavior can contribute to poor prognosis. Although a cancer diagnosis is believed to be a major health threat, patients have different subjective responses to the same diagnosis. All patients with clinical symptoms such as cough, dyspnoea, persistent health status changes and seeking diagnosis of lung cancer are pre-diagnose lung cancer. Individuals who have an abnormal chest radiograph or have symptoms caused by either local or systemic effects of the tumor usually suspect lung cancer. The diagnostic method for lung cancer depends on the type of lung cancer (small cell lung cancer or non-small cell lung cancer [NSCLC]), the size and location of the primary tumor, the presence of metastases, and the patient’s overall clinical status. The poor lung cancer prognosis leads to loss and patient discomfort (Rivera et al., 2013; Vodermaier et al., 2017).

Anxiety is often described as a feeling of worry, fear, or doubt. Anxiety includes feelings or emotions, thoughts, and bodily sensations. Anxiety in lung cancer patients arises from a fear of the future and a fear of self-concept and self-image, a fear of death, the possibility of infertility, a fear of being left behind by a partner, and isolation from social activities. Anxiety can have both physical and psychological effects. Following are some examples of physical anxiety effects: an increase in muscle tension that can cause discomfort and headaches, rapid breathing that can result in dizziness and trembling, and increased blood pressure that can cause disturbances in the heart and kidneys leading to stroke, nausea, and sleep deprivation that can weaken the immune system, making one susceptible to infection. The psychological anxiety effects are irritability, not being able to relax or concentrate, seeing things negatively, and being very pessimistic, and decrease the patient’s quality of life (Sadock et al., 2015).

Therapy in anxious and depressed patients consists of pharmacotherapy and non-pharmacotherapy. Pharmacotherapy for patients with anxiety and depression consists of Selective Serotonin Reuptake Inhibitor (SSRI), Serotonin-Norepinephrine Reuptake Inhibitor (SNRI), tricyclics, benzodiazepine, and pregabalin. In contrast, non-pharmacotherapy consists of cognitive behavior therapy (CBT), supportive psychotherapy, and insight-oriented therapy. CBT is an effective therapy for patients with anxiety disorders and depression. However, further research is needed for its wider use in some patients, particularly in the case of catastrophic diseases (Carpenter et al., 2018).

CBT involves coaching, educating, and promoting constructive behavior. CBT helps patients recognize thinking patterns or mental thoughts and feelings and helps lung cancer patients resolve anxiety and depression to improve quality of life and lung cancer suffering (Basen-Engquist et al., 2003). This study demonstrated the effect of CBT administration on anxiety, depression, and quality of life in pre-diagnostic lung cancer patients.

## Materials and Methods

*Ethical clearance and experimental design*

The research was ethically reviewed and approved by the Moewardi Hospital Committee for Human Subjects Research (Medical) (number 668/VIII/ HREC/2018) (Supplementary 1). This is a randomized controlled trial study. The research was conducted at Dr. Moewardi Surakarta’s hospital. The research subjects were pre-diagnosed lung cancer patients hospitalized at Regional Public Hospital Dr. Moewardi Surakarta from September 2018 to October 2018. They met the criteria for inclusion and exclusion.

*Experimental subjects*

Patients with pre-diagnosed lung cancer who has symptoms are caused by either local or systemic tumor effects or an abnormal radiograph of the chest were given standard therapy. Random assignments was conducted. The inclusion criteria are the following: new patients with pre-diagnosed lung cancer; patients obtaining oral or written consent at the start of the study and signing; and patients meeting the anxiety symptom criteria based on the Hamilton Anxiety Rating Scale (HARS) instrument. This HARS instrument measure the severity of symptoms of anxiety. The scale is made up of 14 items, each of which is defined by a series of symptoms and both psychic anxiety (mental agitation and psychological distress) and somatic anxiety (physical complaints related to anxiety). Each item is scored on a scale of 0 (not present) to 4 (severe), with a total score range of 0-56, where <17 indicates mild severity, 18-24 mild to moderate severity and 25-30 moderate to severe. We measure the depression based on the Hamilton Rating Scale for Depression (HRSD) with interview symptoms of depression experienced over the past week was emphasis on melancholic and physical symptoms of depression. A HARDS included 4 items intended to subtype the depression. a score of 0–7 is generally accepted to be within the normal range (or in clinical remission), while a score of 20 or higher (indicating at least moderate severity).The World Health Organization Quality of Life (WHOQOL-BREF) based quality of life reduction criteria. The exclusion criteria are the following: patients with other cancers, history-taking patients who are known to treat depression and anxiety problems before diagnosing lung cancer because this patients had experience how to reduce their depression and anxiety condition, lung cancer patients with central nervous system metastases, a history of previous substance abuse, head injury, epilepsy, mental retardation, and stroke. The clinical consecutive samples was carried out with the minimum number of samples per group was 16 subjects. Independent variables CBT and Bound: anxiety, depression, and quality of life. Informed consent forms, patient medical records, patient identification details, and assessment sheets for HARS, HRSD, and WHOQOL were used. Patients with pre-diagnosed lung cancer that met the inclusion and exclusion requirements were eligible to join the research study.

*Data analysis*

The data obtained were tabulated and analyzed to determine the effect of CBT on anxiety and quality of life in patients with pre-diagnosed lung cancer, and to test each group’s differences using the Mann–Whitney test. The significant difference was p < 0.05. Both statistical analyses used SPSS 21. 

## Results

*Characteristics of subjects*

This study was conducted on 32 pre-diagnosed lung cancer patients. They were given standard therapy and CBT adjunct therapy at Regional Public Hospital Dr. Moewardi Surakarta. There was no significant distinction within the basic characteristics of the subjects by age. The mean age of the control group in the treatment group is 53.50 ± 15.39 years, with an average age of 57.44 ± 10.29 years. The independent t-test results gave a p-value of 0.402 (p > 0.05). It also shows no significant difference by sex. The majority of patients in the treatment group were male: 9 patients (56.3%). In the control group, most patients were also male: 11 patients (68.8%). The chi-square test results provided a p-value of 0.465 (p > 0.05). There was no significant differences between the treatment and the control groups in the basic characteristics of research subjects; hence, the basic characteristics were homogeneous (Table 1). 

*Effect of CBT in reducing anxiety based on the HARS instrument compared with control*

**Figure 1:**
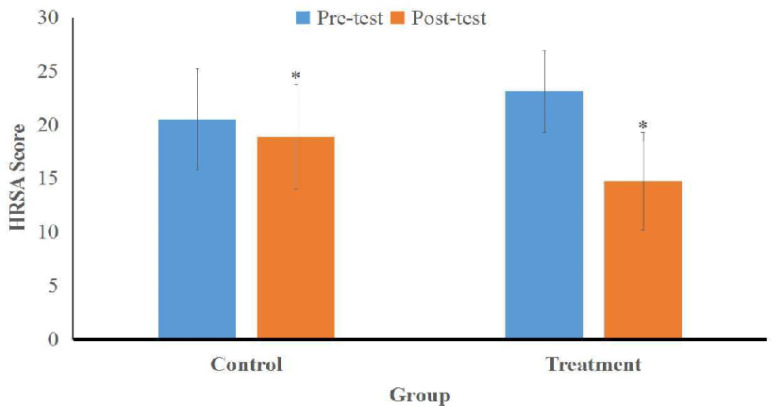
Comparison of HRSA Scores between Treatment and Control Group in the Anxiety Reduction. An asterisk (*) indicates a significant difference between the group in the same test

Effect of CBT in anxiety reduction shows different significantly after the tests conducted. The treatment group (with additional CBT therapy) experienced a reduction in HRSA scores more than the control group (standard therapy). The mean HARS pre-test score in the treatment group was 23.13 ± 3.83 (moderate anxiety) and in the control group was 20.50 ± 4.70 (moderate anxiety), with a p-value of 0.106 (p > 0.05). The mean post-test HARS score was 14.75 ± 4.55 (mild anxiety) in the treatment group and 18.88 ± 4.86 (mild anxiety) in the control group, with a p-value of 0.019 (p < 0.05). The difference in score change HARS post-test−pre-test was −8.38 ± 2.90 or 36.2% in the treatment group and an average of −1.63 ± 3.10 or 8.0% in the control group, with a p-value of 0.000 (p < 0.05) (Table 2; Figure 1).

*Effects of CBT in reducing depression based on the HRSD instrument compared with control (standard therapy)*

**Figure 2:**
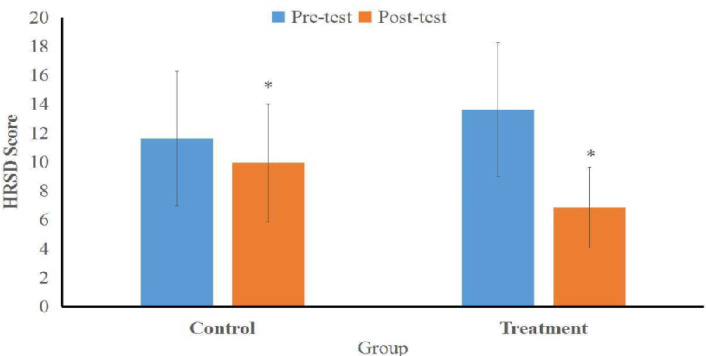
Comparison of HRSA Scores between Treatment and Control Group in the Depression

Effect of CBT in depression reduction also shows different significantly after the tests conducted though control and treatment groups did not find a significant difference. Effect of CBT in reducing depression shows no statistically significant difference between the treatment group and control group before treatment. The pre-test HRSD score in the treatment group was 13.63 ± 4.62 (mild depression) and the control group was 11.63 ± 4.67 (mild depression), with a p-value of 0.233 (p>0.005). The HRSD post-test score was 6.88 ±2.78 (normal) in the treatment group and an average of 9.94 ± 4.06 (mild depression) in the control group with a p-value of 0.007 (p < 0.05) (Table 3, Figure 2).

*Effects of CBT in improving quality of life based on the WHOQOL-BREF instrument compared to control (standard therapy)*

Effect of CBT in life quality improvement exhibited a significant difference in all domain (physical health, psychological health, social relations, and environment). Changes to the WHOQOL-BREF score post-test−pre-test domain 1 (physical health) in the treatment group underwent an average increase of 17.19 ± 11.75 or 16.1% and an increase of 1.55 ± 8.69 or 1.3% in the control group, with a p-value of 0.000 (p < 0.05). It was also similar in domain 2 (psychological health) in which showed an average increase of 16.02 ± 9.41 or 19.6% in the treatment group and an average increase of 2.33 ± 2.33 or 2.6% in the control group, with a p-value of 0.000 (p < 0.05). Changes to WHOQOL-BREF post-test−pre-test domain 3 (social relations) experienced an average increase of 29.69 ± 8.69 or 89.4% in the treatment group and an average decrease of −0.78 ± 4.49 or 2.0% in the control group, with a p-value of 0.000 (p < 0.05). Lastly, domain 4 (environment) had an average increase in both control and treatment group with the value of 0.00 ± 14.07 (0.0%) and 16.80 ± 10.13 (13.7%), respectively with p-value of 0.000 (p < 0.05) (Table 4).

**Table 1:** Characteristics of Research Subjects

Characteristics	Group		p-value
	Treatment (n = 16)	Control (n = 16)	
Age (years)	53.50 ± 15.39	57.44 ± 10.29	0.4021^1^
Gender			0.4652^2^
Male	9 (56.3%)	11 (68.8%)	
Female	7 (43.8%)	5 (31.3%)	

Notes: ^1^, independent t-test (normally distributed numerical data); ^2^, chi square (nominal categorical data)

**Table 2:** Effect of CBT on Reduction of Anxiety

HRSA	Group		p-value
	Treatment	Control	
Pre-test ^1^	23.13 ± 3.83	20.50 ± 4.70	0.106 ^2^
Post-test ^2^	14.75 ± 4.55	18.88 ± 4.86	0.0191^1^
Difference ^2^	8.38 ± 2.90	1.63 ± 3.10	0.0002^2^

Notes: ^1^, Mann–Whitney test (numerical data are not normally distributed); ^2^, independent t-test (normally distributed numerical data); asterisk (*) indicates significantly

**Table 3:** Effect of CBT in Reducing Depression

HRSD	Group		p-value
	Treatment	Control	
Pre-test ^1^	13.63 ± 4.62	11.63 ± 4.67	0.233^1^
Post-test ^2^	6.88 ± 2.78	9.94 ± 4.06	0.0072^ 2^
Difference ^2^	−6.75 ± 3.30	−1.69 ± 3.42	0.0001 ^1^

Notes: ^1^, independent t-test (normally distributed numerical data); ^2^, Mann–Whitney test (numerical data are not normally distributed); asterisk (*) indicates significantly different between the groups (p<0.05).

**Table 4:** Effects of CBT on Improving Quality of Life

WHOQOL-BREF	Group		p-value
	Treatment	Control	
Domain 1 (physical health)			
Pre-test	107.03 ± 18.94	115.25 ± 17.37	0.2111^1^
Post-test	124.22 ± 15.79	116.80 ± 18.92	0.2381^1^
Difference	17.19 ± 11.75	1.55 ± 8.69	0.0002^2^
Domain 2 (psychological health)			
Pre-test	81.64 ± 11.96	89.86 ± 17.95	0.0702^2^
Post-test	97.66 ± 14.77	92.19 ± 19.30	0.3751^1^
Difference	16.02 ± 9.41	2.33 ± 2.33	0.0002^2^
Domain 3 (social relations)			
Pre-test	33.20 ± 8.14	38.28 ± 9.09	0.1482^2^
Post-test	62.90 ± 1.56	37.50 ± 9.13	0.0002^2^
Difference	29.69 ± 8.69	-0.78 ± 4.49	0.0002^2^
Domain 4 (environment)			
Pre-test	123.05 ± 16.25	133.59 ± 19.88	0.1111^1^
Post-test	139.84 ± 12.05	133.59 ± 23.48	0.3511^1^
Difference	16.80 ± 10.13	0.00 ± 14.07	0.0002^2^

Notes: ^1^, independent t-test (normally distributed numerical data); ^2^, Mann–Whitney test (numerical data are not normally distributed). asterisk (*) indicates significantly different between the groups (p<0.05).

**Figure 3:**
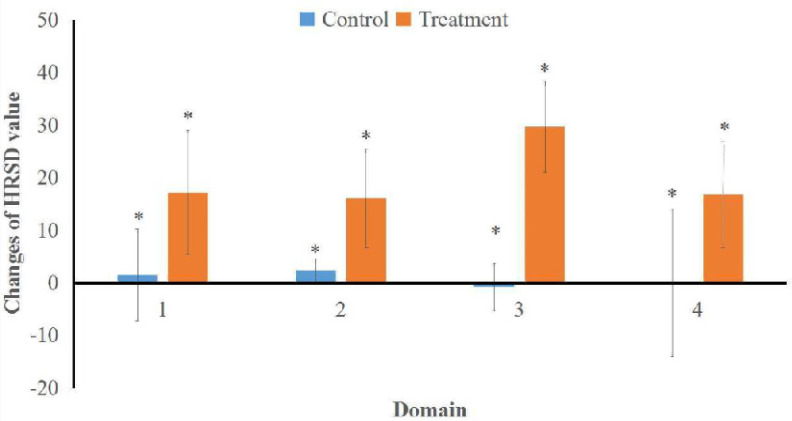
Comparison of Difference in Changes WHOQOL-BREF Score between the Treatment Group and the Control Group. An asterisk (*) indicates the changes were significantly different between control and treatment groups

## Discussion

The difference in post-test − pre-test HARS score change decreased by 36.2% in the treatment group and decreased by 8.0% in the control group with a p-value of 0.000 (p < 0.05). This means that therapy adjuvant CBT psychotherapy intervention given for as many as 6 sessions every 2 days is effective in reducing anxiety rates.

Anxiety is the body’s normal reaction to actual or potential threats. The body then responds to brace for threat by combining chemical and physical changes, called stress responses. Anxiety is mediated by two systems of neurochemical mechanisms and neuropeptides, which have an impact on the brain and subcortical cortical region associated with anxiety disorder symptoms.

This brain region relates to actions of stress, anxiety, and anxiety behavior. Anxiety-related GABA receptors affect serotonin and norepinephrine, antagonists of neurotransmitter systems such as corticotropin-releasing factor and substance P, minimize glutamate neurotransmission, such as metabolic glutamate receptors, and promote neurotrophic factors that tend to increase neurogenesis (Gilhotra and Dhingra, 2010).

The incidence of subjective anxiety has the characteristics of two components (physical and emotional), which affect the cognitive process of lung cancer patients. Heart palpitations, anxious feelings, fear, anxiety, insomnia, cold or sweaty palms, shortness of breath, inability to calm down, dry mouth, numbness in the hands or feet, nausea, muscle weakness, and headache are common symptoms and signs of anxiety. Anxiety-influencing factors include predisposing factors, cancer anxiety, disease and treatment factors, and aggravating symptoms. Lung cancer patients undergo coping strategies, including avoidance, denial, and attempts to increase health. Monitoring involves identifying risks and a growing understanding of the symptoms often seen in nervous patients with lung cancer (Soodan and Arya, 2015).

Our study revealed that the difference in score change post-test−pre-test HRSD decreased by 49.5% in the treatment group and decreased by 14.5% in the control group, with a p-value of 0.000 (p < 0.05). This means that the therapy adjuvant CBT psychotherapy intervention given for as many as 6 sessions every 2 days effectively reduces the levels of depression.

For some patients with lung cancer, mental health issues such as anxiety, depression, post-traumatic stress disorder, adjustment disorders, social phobia, and organic mental disorders typically arise. Lung cancer stigma (LCS) is a health-related stigma triggered by misleading views of the smoking–lung cancer causal relationship. LCS leads to high stress in patients with lung cancer and can help explain the poor quality of life. Previous research found that LCS correlated with higher depression, lower quality of life, and higher anxiety (Brown et al., 2014; Yi and Syrjala, 2017).

Aaron T. Beck and Albert Ellis described the core concepts of CBT in 1960. CBT became the most empirically supported psychotherapy treatment with more than 300 controlled trials supporting the efficacy of cognitive therapy, as mentioned by Judith Beck. In the same way, Mahoney’s behavioral therapist in 1974 noted that behavioral therapy has limitations in explaining and treating certain patients. At the same time as Beck developed a cognitive therapy approach to depression, Seligman and Abramson in 1979 experimented with the theory that negative explanatory methods increase the risk of depression. Thus, the hypothesis stating that “There is an effect of giving CBT to repair depression” is proved (Butler et al., 2006; Chand et al., 2020).

The results showed that the difference in WHOQOL-BREF score change post-test−pre-test increased by 16.1% in treatment group domain 1 (physical health), increased by 19.6% in domain 2 (psychological health), increased by 89.4% in domain 3 (social relations), and increased by 13.7% in domain 4 (environment). In the control group, domain 1 increased by 1.3%, domain 2 increased by 2.6%, domain 3 decreased by 2.0%, and domain 4 increased by 0.0%, where the WHOQOL-BREF score on each domain had a p-value of 0.000 (p < 0.05). This means that the therapy adjuvant CBT psychotherapy intervention given for as many as 6 sessions every 2 days improves the quality of life. Quality of life is a person’s perception of the cultural context and norms appropriate to the person’s place of life and related to the goals, expectations, standards, and concerns throughout the person’s life (WHO, 2004).

The side effects of surgical therapy include long-term pain that can be felt for several weeks and cause psychiatric aspects due to the inability to heal psychologically and physiologically within a few months. The quality of life of patients, on average, has improved in the third, sixth, and ninth months after surgery. Mortality in lobectomy is 1%–2%, and in pneumonectomy is 6%–7%. Mortality increases with age, comorbid disease, extent of resection, and respiratory complications. Patients who undergo surgery undergo post-operative complications as much as 30%–40% (Li and Kern, 2015).

*Limitation*

The limitation of this research was using the open trial method; it can affect the results of the study because researchers and research subjects know the same interventions given. The resilience factors of the subjects (cognitive skills, psychological resources, and social support) can affect the bias at the end of the study.

In conclusion, CBT has an effect on the improvement of anxiety, depression and quality of life in pre-diagnose lung cancer patients.

## Author Contribution Statement

All authors contributed equally to this work.

## Ethical Approval

This research has been approved by Health Research Ethics Committee of Dr. Moewardi General Hospital, School of Medicine Sebelas Maret University, No.668/VIII/HREC/2018.

## Conflict of interest

All authors declare no conflict of interest.
